# Supplementary material for: Identification of photoperiod-induced specific miRNAs in the adrenal glands of Sunite sheep (Ovis aries)
Source: Front Vet Sci. 2022 Jul 22;9:888207. doi: 10.3389/fvets.2022.888207 (PMC9354845; doi:10.3389/fvets.2022.888207)
Supplement: Supplementary file 1 [file Data_Sheet_1.ZIP › Supplementary file/miRDeep2 file.zip!/miRDeep2 file/Novel_Structure/LP42A1/Novel_208.pdf]

Provisional ID	:	NC_019458.2_379
Score total	:	2.4
Score for star read(s)	:	-1.3
Score for read counts	:	0
Score for mfe	:	2.1
Score for randfold	:	1.6
Score for cons. seed	:	
Total read count	:	12
Mature read count	:	11
Loop read count	:	0
Star read count	:	1

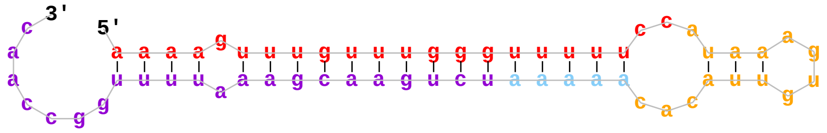

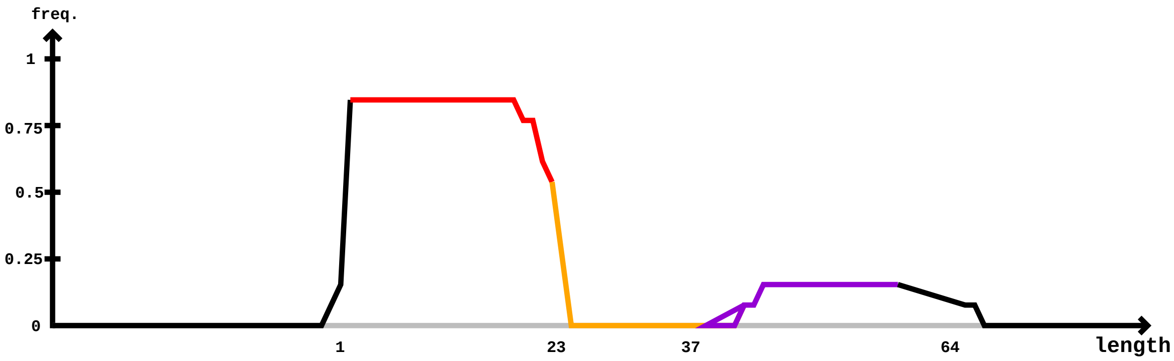

Mature Star

```
5'- gaaaccugcuuguauguauuggguuggccaaaaguuuguuuggguuuuuccauaaaguguuacacaaaaaucugaacgaaauuuuggccaacccaauauaugaagagagaag   -3'    obs
    gaaaccugcuuguauguauuggguuggccaaaaguuuguuuggguuuuuccauaaaguguuacacaaaaaucugaacgaaauuuuggccaacccaauauaugaagagagaag          exp
    .....((.(((.(((((((((((((((((((((.(((((((((((((((...(((....)))...))))))))))))))).)))))))))))))))))))))..)))))...   reads      mm         sample
    ...........................cAaaaaguuuguuuggguuu.................................................................   1          1          seq
    ............................caaaaguuuguuuggguuuuu...............................................................   1          0          seq
    .............................aaaaguuuguuuggguuuuu...............................................................   1          0          seq
    .............................aaaaguuuguuuggguuuuuc..............................................................   1          0          seq
    .............................Gaaaguuuguuuggguuuuucc.............................................................   6          1          seq
    .............................aaaaguuuguCuggguuuuucc.............................................................   1          1          seq
    ......................................................................ucugaacgaaauuuuggccaac....................   1          0          seq
    ........................................................................ugaacgaaauuuuggccaaccca.................   1          0          seq
```
